# Copper Homeostasis is influenced by Ics3 in *Saccharomyces cerevisiae*

**DOI:** 10.17912/micropub.biology.001801

**Published:** 2025-10-27

**Authors:** Lucas Tang, Aseel AlKaabi, Damon Meyer

**Affiliations:** 1 Western University of Health Sciences, Lebanon, Oregon, United States; 2 College of Health Sciences, California Northstate University, Rancho Cordova, California, United States

## Abstract

*Saccharomyces cerevisiae* has several unique open reading frames that contribute to overall fitness. This study examined *ICS3* , a contributor of Cu ^2+^ homeostasis. Our results successfully replicate previous work by the Monteiro laboratory using a different method, confirming the positive effect of *ICS3* on maintaining copper homeostasis for cell growth. No significant difference in growth of *ics3Δ* mutants was observed for Zn ^2+^ , another oxidative stressor. In addition, *MATa* and *MATα* mating types did not influence sensitivity to Cu ^2+^ or Zn ^2+^ .

**Figure 1. Cell survival following exposure to metals:**
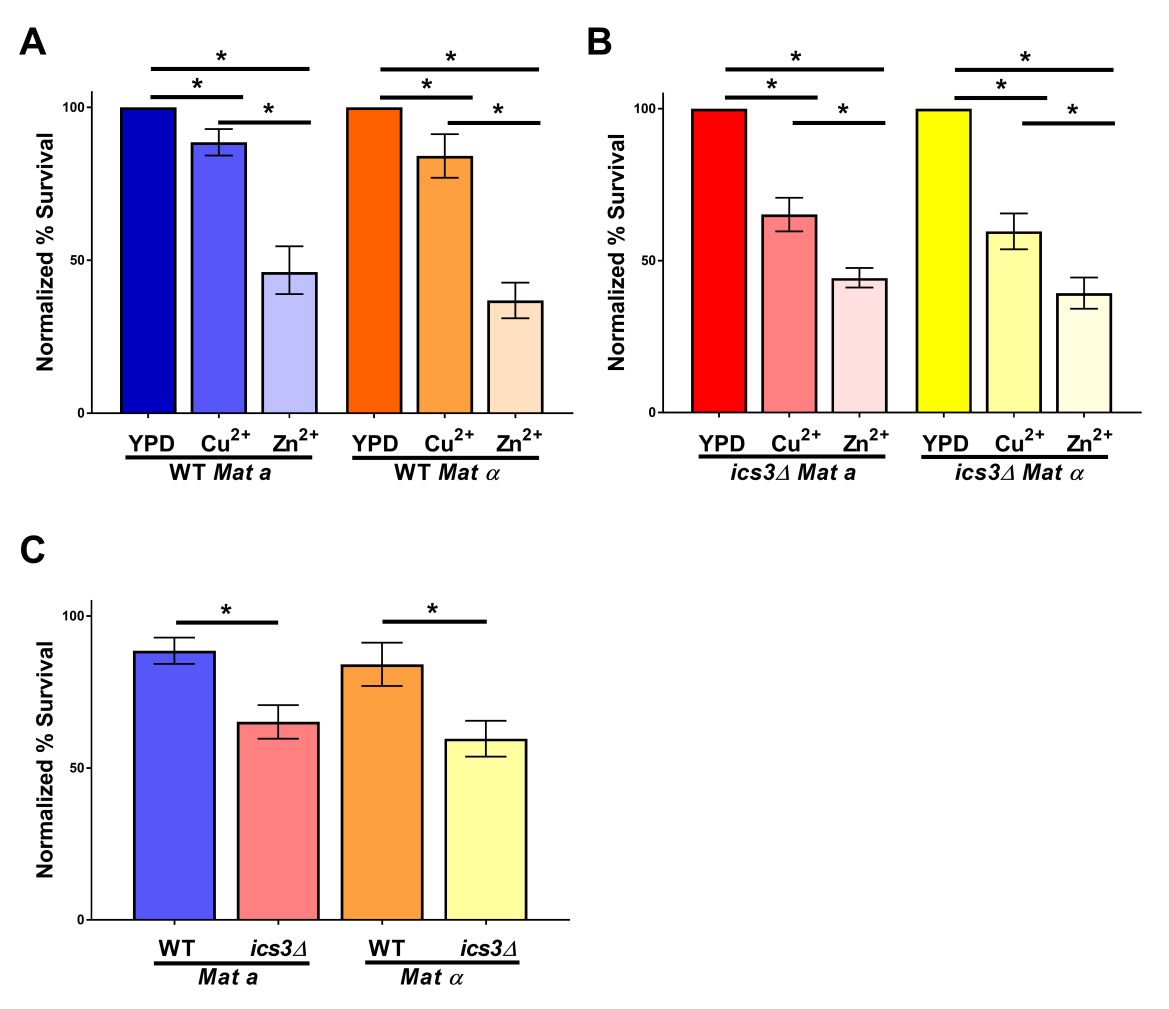
Cell survival of wildtype (WT) and *ics3Δ* mutants in 10 mM CuSO _4_ (Cu ^2+^ ) or 10 mM ZnSO _4_ (Zn ^2+^ ) was normalized to the number of colony forming units on YPD (# colonies on Cu²⁺ or Zn²⁺/# colonies on YPD × 100). (A) Cell survival of WT *MATa* and *MATα* haploids following growth in YPD, Cu ^2+^ or Zn ^2+^ . (B) Cell survival of *ics3Δ* mutant *MATa* and *MATα* haploids following growth in YPD, Cu ^2+^ or Zn ^2+^ . (C) Direct comparison of WT and *ics3Δ* mutants exposed to Cu ^2+^ in *MATa* and *MATα* from panels A-B. (A-C) Data shown are the mean ± 95% confidence interval from a minimum of 12 independent cultures. Asterisks indicate statistical significance (p < 0.05) following a student t-test.

## Description

*Saccharomyces cerevisiae* is a model organism commonly used for genetic study due to ease of manipulation. In addition to the known 6,000 annotated open reading frames (ORFs) that are part of the canonical genome translated into proteins (Goffeau et al., 1996) , non-canonical, novel protein coding genes thought to have arisen through *de novo* gene birth have been identified as contributing to organism fitness (Carvunis et al., 2012; Wacholder et al., 2023). First named in a high throughput study by Entian et al. (1999) as an ORF of unclear function, *ICS3* has since been associated with oxidative stress homeostasis, specifically with copper homeostasis (Alesso et al., 2015). Cu ^2+^ ions are necessary for cell growth in *S. cerevisiae* in trace amounts and can quickly become toxic as an oxidative stressor producing reactive oxygen species — demanding the need for tight cellular regulation of homeostasis (Culotta et al., 1995). Elucidating the role of an uncharacterized ORF in such a biochemical pathway can inform the role of non-canonical ORFs and *de novo* gene birth in organism fitness as proposed by Carvunis et al. (2012). This study follows up on the research by Alesso et al. by using a different methodology to evaluate cellular viability of wildtype and *ics3Δ* mutants in the presence of increasing amounts of copper and zinc.

Alesso et al. (2015) reported expression of *ICS3* to be vital towards cell survival in the presence of excess copper. This was tested using a strain where *ICS3* was deleted in the BY4741 background (Alesso et al., 2015). In the current study, a quantitative analysis of individual colony-forming units (CFUs) exposed to copper was performed in the same BY4741 background in an attempt to complement the findings of Alesso et al. (2015). The colony formation assay was used since it is a robust measure of individual cell reproductive potential while the serial dilution spot assay used by Alesso et al. (2015) provides an estimate of growth and survival that is less precise. Experiments in this study involved *MATa* and *MAT𝛼* haploid wildtype and *ics3Δ* mutants exposed to 10mM of CuSO _4_ in liquid YPD for four hours then plated onto solid YPD medium and incubated for 48-72 hours, after which individual colony counts were recorded. Since previous work indicated that pH influenced growth results in CuSO _4_ , all media was adjusted to a pH of 6.0 (Alesso et al., 2015). Our results showed CuSO _4_ had a significant effect on cell survival that was independent of mating type in both wildtype and *ics3Δ* mutants ( Figure 1A- C). This supports the previous results by Alesso et al. (2015), using another method of measurement, which confirms the role of Ics3 in regulating Cu ^2+^ ions. Therefore, the fitness of *S. cerevisiae* is impacted during acute exposure to environmental copper that can come from natural sources or human activity (Poggere et al., 2023).

In addition to our results with Cu ^2+^ , we wanted to explore if the effects observed are due to a general oxidative stress response to metal cations or is specific to copper homeostasis regulation. To address this, the same experiments were repeated in wildtype and *ics3Δ* mutants when exposed to ZnSO _4_ , which produce Zn ^2+^ ions that are another cationic oxidative stressor similar to Cu ^2+^ . Zn ^2+^ is a bioessential metal for *S. cerevisiae* , playing a role in protein and membrane stabilization (White & Gadd, 1987), and as an essential catalytic component of many enzymes (Eide, 2003). Similarly to Cu ^2+^ , Zn ^2+^ becomes toxic to the cell in excess and is tightly regulated (Eide, 2003). Alesso et al. (2015) found negative results for the effects of Fe ^2+^ and Co ^2+^ as oxidative stressors on cell growth when *ICS3* was deleted. This suggests that Ics3 may function more broadly to protect cells exposed to cationic oxidizers beyond just copper. To test this, the same protocol of a four hour exposure to the experimental stressor Zn ^2+^ was performed on *S. cerevisiae* cells in YPD with 10mM ZnSO _4_ , followed by plating, and incubation for 48-72 hours before colony numbers were counted. Although overall growth was significantly decreased following the four hour exposure to zinc compared to when cells were only in YPD, this was observed for both the wildtype and *ics3Δ* mutants that were independent of mating type ( Figure 1A- C). Interestingly, a significant decrease in cell survival was observed in cells exposed to Zn ^2+^ compared to Cu ^2+^ across all genotypes examined ( Figure 1A- C).

While cell survival in both wildtype and *ics3Δ* mutants was affected by exposure to Cu ^2+^ and Zn ^2+^ , the specific contribution of Ics3 in protecting cells after Cu ^2+^ exposure required a direct comparison between wildtype and *ics3Δ* mutants. Analysis across both *MATa* and *MATα* , the same significant decrease in cell survival in *ics3Δ* mutants exposed to Cu ^2+^ when compared to wildtype was observed ( Figure 1C ). In contrast, exposure to Zn ^2+^ did not show a differential effect in wildtype compared to *ics3Δ* mutants ( Figure 1A- C).Therefore, it is likely the Ics3 protein does not contribute towards cell fitness in the presence of Zn ^2+^ and is more selective in which cationic oxidative stressors it regulates. Taken together, our results support Alesso et al’s (2015) findings by showing a specific role of Ics3 in protecting cells from the toxic effects of copper exposure.

The literature discussing the function of Ics3 in the cell has so far only considered Cu ^2+^ (Entian et al., 1999; Alesso et al., 2015), Co ^2+^ and Fe ^2+^ (Alesso et al., 2015). This study supports the role of Ics3 in Cu ^2+^ homeostasis, which is in alignment with previous studies, but also provides evidence that Ics3 does not play a role in Zn ^2+^ homeostasis, suggesting it can discriminate against specific cationic oxidizers. Information from the LoQAtE database, which provides protein localization information under various stress conditions, suggests that the Ics3 protein localizes to the mitochondria (Breker et al., 2013). However, additional experiments would be useful to specifically locate its position in the mitochondria, which has yet to be answered. This evidence that the Ics3 protein localizes to the mitochondria (Breker et al., 2013), combined with reports of the mitochondria regulating Mn ^2+^ homeostasis (Bleackley & MacGillivray, 2011; Reddit et al., 2009), indicate that further investigation is required to determine if Ics3 is truly limited to Cu ^2+^ homeostasis regulation. Potential future studies could include other bioavailable transition metal cations that are oxidative stressors and regulated via the mitochondria (Stoiber, 2010).

## Methods

All strains used in this study were isogenic to the BY4741 or BY4742 background. Wildtype and *ics3Δ* mutants were grown in liquid YPD, liquid YPD supplemented with 10mM CuSO _4_ , or liquid YPD supplemented with 10mM ZnSO _4_ . Following the protocol described by Alesso et al. (2015) an overnight starter culture of wildtype or *ics3Δ* mutant cells were diluted into fresh media (YPD, YPD with 10mM CuSO _4_ , and YPD with 10mM ZnSO _4_ ) to an initial OD _600_ of 0.4. Cultures were incubated for four hours in liquid media with or without metal cation before making an appropriate dilution and plating to YPD. Cells were incubated for 48-72 hours until colonies were large enough to be counted. Survival on 10mM CuSO _4_ or 10mM ZnSO _4_ was normalized to growth on YPD by taking the total colony count from a metal exposed culture and dividing by the total colony count from YPD without metal and displayed as a percent (%). Statistical analysis was performed using Microsoft Excel and data visualization using GraphPad Prism.

## Reagents

**Table:** 

Strain	Genotype	Source
BY4741	*MATa his3Δ1 leu2Δ0 met15Δ0 ura3Δ0*	California Northstate University, College of Health Sciences, Rancho Cordova, California, 95670.
BY4742	*MATα his3Δ1 leu2Δ0 lys2Δ0 ura3Δ0*	The University of Pittsburgh, School of Medicine, Department of Computational and Systems Biology, Carvunis Lab
yCHS90	*MATa his3Δ1 leu2Δ0 met15Δ0 ura3Δ0 ics3Δ*	The University of Pittsburgh, School of Medicine, Department of Computational and Systems Biology, Carvunis Lab
yCHS91	*MATα his3Δ1 leu2Δ0 lys2Δ0 ura3Δ0 ics3Δ*	The University of Pittsburgh, School of Medicine, Department of Computational and Systems Biology, Carvunis Lab
Reagent	Final Concentration	Company
Copper (II) Sulfate	10 mM	VWR
Zinc (II) Sulfate	10 mM	VWR
